# The London Post-Graduate Course at the Paddington Infirmary

**Published:** 1890-10-18

**Authors:** 


### The Practitioner's Mirror and Retrospect.

[*The Editor will be glad to receive offers of co-operation and contributions from members of the profession. There is no doubt that much valuable material full of interest and instruction is at present lost to science. This is largely so in the case of* (1) *the younger and less-known members of the hospital staff* (2) *those connected with cottage hospitals, and* (3) *the great body of medical practitioners not attached to any institution. The co-operation of all is cordially invited to enable the Editor to make* The Hospital *of the utmost possible use and value to the profession. Specialists willing to review books on their special subjects are invited to communicate this fact at once. All letters should be addressed to* The Editor, The Lodge, Porchester Square, London, W.]

### THE LONDON POST-GRADUATE COURSE AT THE PADDINGTON INFIRMARY.

In the number of The Hospital issued on August 23rd, wo published an account of the Metropolitan Workhouses and Infirmaries, with especial reference to the resolution of the Paddington Board of Guardians, which ran as follows:— "That Professor J. Hutchinson and Dr. Broadbent be invited to examine the cases in the infirmary in consultation with the medical superintendent, and to deliver lectures at the infirmary in connection with the London Post-Graduate course now in process of formation, it being understood that such a procedure is not attended with additional cost to the ratepayers, and does not interfere with the existing management and discipline of the institution."

In this article it was pointed out that the Poor Law or Workhouse Infirmaries of the Metropolis have together 12,170 beds, and that the hospitals together 9,173 beds, also, that the former are occupied chiefly by medical and the latter chiefly by surgical cases. Notwithstanding the enormous amount of material that exists in the infirmaries, they are practically closed to the profession at large. There are no students to criticise the medical officers' methods of diagnosis and treatment, and keep them up to their work; no clinical clerks and dressers to help them to investigate and treat the patients; no consultants to advise in cases of difficulty. These great storehouses of disease are closed to the outer world. The medical staff of these institutions has consisted until quite recently of only two medical men, one of whom has also the general administrative control of the establishment in addition to his regular medical duties. Thus these gentlemen, in one infirmary, have under their control some 253 beds each. How is it possible that they can do their full duty to all these patients under their care?

On Monday last the ex-President of the Royal College of Surgeons gave the opening lecture to the London Post-Graduate course at the Paddington Infirmary. The substance of this address, with his kind permission, we give below. On the list of lecturers now lying before us for the infirmary section of the course we see the names of Drs. Bristow, Cheadle, and Savill, together with Messrs. Reginald Harrison and Frederick Treves.

We are thus very pleased to see that the authorities are beginning to recognise the importance of opening these institutions for the purpose of teaching.

### Diseases Common to Old People.

Mr. Jonathan Hutchinson, in commencing his lecture, said that as the infirmary abounded in cases illustrating many forms of senile complaints, he proposed taking for his subject senile diseases, or, rather, diseases common to old people.

Before beginning the subject of his lecture proper, he mentioned a case of strangulated hernia which had become cured, although left to itself. The case was that of an old woman who was being treated for colic. One day she found that there was a swelling about the femoral region which was discharging fœcal material. In the course of time this quite healed up—that is, she had a strangulated femoral hernia which had become healed. Some time afterwards the patient died from bronchitis, and on *post-mortem* examination the gut was found to be closely adherent all about the femoral region. He had just seen a similar case. A patient was then shown who had been admitted into the infirmary with a swelling resembling an abscess in the groin; this was opened and fœcal matter came out. The sinus had now quite healed, and the patient had no symptoms, the bowels were opened regularly. It was said that in these cases probably only some segment, and not the whole calibre of the gut, was involved in the hernia.

The two skulls that were shown at the University College Medical Society, with hyper- and exostoses over the regions of bone supplied by branches of the fifth nerve, and which we report on another page with the substance of the address before this society, were also exhibited.

Mr. Hutchinson now proceeded to the subject of his lecture, "senility," and he commenced with the skin, which he said underwent atrophy. So also the glands, and even the glandular appendages of the mucous membranes become atrophied. Thus the skin becomes translucent, so that the tendons lying below could be very clearly seen. In connection with this subject he referred to a picture of Millais of an old lady which showed this condition very beautifully, and he thought the painting should have been purchased by one of the colleges as a specimen illustrating a pathological point. Passing to the bones he referred to the frequency of fractures of the neck of the femur in old people, also mentioning that in these cases union does not always occur. If anything happened to this region in children it was possibly a separation of the epiphesis, and during adult life fracture [of this region was exceedingly rare, but in old age it was a very common occurrence. He said that probably the same process occurred in the case of all the bones of old people, but in that they were rarely exposed to the same risks of the younger ones, fracture of other bones was not common. The explanation was that there was an absorption especially of the outer and harder parts of the bone. So also in the case of the skull, the bones were subject to a double process. As age advances the brain shrinks, becomes harder and smaller, and its place is taken partly by fluid and partly by an increase of bone from the inner table of the skull, and was sometimes attended by absorption from the outer table; hence the head as a whole becomes smaller, and there may be an actual thinning of the calcarium.

He then returned to the skin, and spoke of the tendency to the formation of outgrowths. The explanation of many of these was, that in old people inflammation is attended with but little hyperemia; there is a general tendency of all inflammatory processes to become chronic, and often extremely so. As a result of this there was a great tendency to local growths in old people. In illustration of these remarks some diagrams and drawings were shown of senile warts, &c.; also some illustrating the fact that old people were very liable to small localised papillomatous growths, and these have a tendency to become black. This colouration was not due to pigment, but dirt, and was especially liable to occur on the growths over the back. The distinction between these and molluscum fibrosum which sometimes becomes more conspicuous in old people was pointed out. The next point discussed was that of "senile freckles." That freckles were common in some children was generally known. Mr. Hutchinson believed that this was especially the case when one parent was dark and the other one fair. Freckles in children occurred scattered over the whole face, whereas in old people they occurred especially about the eyelids, and often they were not symmetrical; again, they were not of the rich brown colour of childhood, but frequently black, and might possibly be mistaken for melanoses. The subject of cancer was next discussed, and its association often with papilloma and freckles mentioned. Both papilloma and freckles were probably due to nutritive changes, and many cases of cancer were associated with these smaller changes. Senility gave certainly a very strong proclivity to the formation of cancer, and some reference was made to the lower animals, but few of which were kept till they were very old; but if they were kept long enough, they frequently died from cancer, at least it was so in the case of dogs. If cancer occurred before middle life it was probably inherited from an ancestor who had acquired it late in life. Great stress was laid on the fact that it was not that these warts and like structures necessarily become cancerous, but that they might do so. With regard to the statement that cancer occurs in old people, it must be remembered that some parts of the body grow old before others; the mammary gland in the female grows old long before the rest of the body. According to these views cancer was not due to the introduction of any special material into the system; there was nothing specific in its nature; it was simply a modification of what occurs in chronic inflammation. It sometimes happened that multiple cancerous growths occurred in very old people. In some, all stages of the process might be seen in the same individual, between simple chronic inflammatory and cancerous processes, so that it would be impossible to say which were cancerous and which were not.

Another disease of old men, namely, enlargement of the prostate gland, was then spoken of. This occurred only in old persons or in younger people prematurely aged. It was not necessarily a disease of senility; it did not occur in all old people.

The occurrence of prostatic disease prevented people from living to extreme old age, and hence it was not found in the very aged; in fact, it in one way or another cuts short the life of many a healthy old man.

The last subject treated was the disease of the vessels. A true calcification of the arteries produced the condition of so-called knotted cords; although this condition was associated with old age, many old people never got calcification of their arteries (Humphry). The calcification might give rise to rupture of the vessel and apoplexy, and also to senile gangrene.

Another form of degeneration of vessels often occurred, namely, hypertrophic dilatation, and this condition might give rise to aneurysms,' which were sometimes symmetrical. It was considered possible that this condition might be confined to certain limbs, such as the upper or the lower extremity. So also the tendency for dilatation of the veins and of thrombotic phlebitis was mentioned.

After the lecture Mr. Hutchinson went up into the wards, and showed there some cases illustrating the subject of his discourse. Many drawings and paintings were also shown of cases of cancer, among which were some illustrating what is known as "cratiform" cancer, a very rapid variety, of malignant growth.

Pathology in Workhouse Infirmaries.

Dr. Savill, the medical superintendent of the infirmary, took as the subject of his lecture "The Study of Pathology in Workhouse Infirmaries." He stated that there were three kinds of pathology: 1. Laboratory, that is to say, experimental. 2. Dead-house, that is, morbid anatomy; and 3. Ward, that is, clinical pathology. He next spoke of the extent of the field in the infirmaries, and said there are some 12,170 beds in the metropolitan area as compared to 9,174 beds in the hospitals. Thus they were the most general of general practitioners, having every possible variety of medical and surgical work, together with much special administrative.

The kind of cases met with by the workhouse doctor were then discussed and compared with those found in the general hospitals. The hospital cases being selected ones for acuteness, severity, or curability, the infirmary, on the other hand, took in everyone who was ill and destitute. So, also, they met with several special forms of disease, such as those of senile decay, very chronic rheumatism, many forms of nerve disease, including hysteria in both sexes. During last year they had 30 cases of this complaint in the wards and yet some people thought that hysteria was only a disease of the richer classes, who have more time to think about it. Again they were able to follow the cases to the end.

Dr. Savill then referred to a very interesting case of arterio-fibrosis, he had watched for some years. The case was one uncomplicated with cardiac disease or renal mischief, but suffered much from giddiness. One day the patient had an attack of apoplexy and died.

He also described the case of D—— D——, a man, aged thirty, a stableman, rather a free drinker; had a winter cough for three years. He was admitted in 1879 with dilatation of the right side of the heart, general bronchitis, and œdema of the legs, with no albumen. On December 18th he had a series of four epileptiform fits, was convulsed especially on the left side; he vomited and passed his urine involuntarily.

On recovering from these the patient stated he had never had a fit before in his life. Shortly after this he left the infirmary, but on August 23rd of this year he returned, very short of breath and with a very rapid pulse; he was admitted, but died within two hours. Shortly before death he made a considerable noise, and foamed at the mouth.

Post-mortem: There was a little fluid in the pleuræ, the lungs showing much bronchitic and emphysematous changes. The heart weighed 20 ounces, and was very fatty, the right side much dilated, the cardiac substance was half fat and half degenerated muscle, the valves and the aorta were healthy. The liver was also very fatty, and weighed 72 ounces. The brain was very pale, and although sections were examined microscopically, no structural changes were to be found. He was thus led to believe that the epileptiform fits were due to an anæmia of the brain substance, rather than to what is generally understood by epilepsy. Specimens, both macro and microscopical, were then shown, sections of the fatty organs being stained with osmic acid.

